# Mechanism of Chinese botanical drug Dizhi pill for myopia: An integrated study based on bioinformatics and network analysis

**DOI:** 10.1097/MD.0000000000034753

**Published:** 2023-09-22

**Authors:** Longkun Liu, Yoann Birling, Yan Zhao, Wenxin Ma, Yang Tang, Yuxin Sun, Xuehui Wang, Mingkun Yu, Hongsheng Bi, Jian-ping Liu, Li Li, Zhaolan Liu

**Affiliations:** a School of Traditional Chinese Medicine, Beijing University of Chinese Medicine, Beijing, China; b NICM Health Research Institute, Western Sydney University, Penrith, NSW; c Beijing University of Chinese Medicine Third Affiliated Hospital, Beijing University of Chinese Medicine, Beijing, China; d Center for Evidence-Based Chinese Medicine, Beijing University of Chinese Medicine, Beijing, China; e Shandong University of Traditional Chinese Medicine, Shandong, China; f Affiliated Eye Hospital of Shandong University of Traditional Chinese Medicine, Shandong, China; g Beijing Institute for Drug Control, NMPA Key Laboratory for Safety Research and Evaluation of Innovative Drugs, Beijing Key Laboratory of Analysis and Evaluation on Chinese Medicine, Beijing, China.

**Keywords:** Chinese botanical drug, differentially expressed genes, Dizhi pill, molecular docking, network analysis, WGCNA

## Abstract

To identify the active constituents, core targets, immunomodulatory functions and potential mechanisms of Dizhi pill (DZP) in the treatment of myopia. The active constituents and drug targets of DZP were searched in the TCMSP, Herb databases and correlational studies. The targets of myopia were searched in the TTD, Genecards, OMIM and Drugbank databases. Gene expression profile data of GSE136701 were downloaded from the GEO database and subjected to WGCNA and DEG analysis to screen for significant modules and targets of myopia. Intersectional targets of myopia and DZP and core targets of myopia were analyzed through the String database. The GO and KEGG enrichment analyses of the interested targets were conducted. Cibersort algorithm was used for immune infiltration analysis to investigate the immunomodulatory functions of DZP on myopia. Autodock was used to dock the important targets and active constituents. Eight targets (STAT3, PIK3CA, PIK3R1, MAPK1, MAPK3, HSP90AA1, MIP, and LGSN) and 5 active constituents (Quercetin, Beta-sitosterol, Diincarvilone A, Ferulic acid methyl ester, and Naringenin) were identified from DZP. In pathways identified by the GO and KEGG enrichment analyses, “ATP metabolic process” and “AGE-RAGE diabetes complication signaling” pathways were closely related to the mechanisms of DZP in the treatment of myopia. Molecular docking showed that both the intersectional targets and core targets of myopia could bind stably and spontaneously with the active constituents of DZP. This study suggested that the mechanisms of DZP in the treatment of myopia were related to active constituents: Quercetin, Beta-sitosterol, Diincarvilone A, Ferulic acid methyl ester and Naringenin, intersectional targets: STAT3, PIK3CA, PIK3R1, MAPK1, MAPK3, and HSP90AA1, core targets of myopia: MIP and LGSN, AGE-RAGE signaling pathway, positive regulation of ATP metabolic process pathway and immunomodulatory functions.

## 1. Introduction

Myopia is the most common eye disease,^[1]^ and its prevalence has been consecutively increasing in recent years,^[2]^ with some studies predicting that the global myopia population will reach 5 billion by 2050.^[3]^ In addition to affecting the quality of life, severe myopia can further develop into more severe diseases such as glaucoma and cataract, which may even lead to blindness.^[4–6]^ Current treatments for myopia include pharmacotherapy, keratoplasty and laser corneal surgery.^[7]^ However, the specific mechanisms of myopia development and myopia gene host reactions are still unclear.^[2,8]^ Existing drug therapies, atropine eye drops most commonly, have photosensitive, anticholinergic and other side effects,^[9]^ and surgical treatment and laser treatment can cause some invasive side effects such as loss of vision, high myopia and retinal damage.^[10]^ Chinese botanical drugs (CBD) were traditionally used for myopia in China. They are considered relatively safe and noninvasive,^[11]^ and are now widely used in combination with acupuncture to improve myopia in children.^[12]^ Studies showed that CBD could improve retinal lesions and restore visual acuity and visual field.^[13]^

Dizhi pill (DZP) is a CBD formula that was first presented in the Renzhai Zhi Zhi Fang, which mentioned that “Dizhi pill is indicated for the inability to see far away.” It consists of Rehmannia glutinosa (Gaertn.) DC. [Orobanchaceae] (Sheng Dihuang), Asparagus cochinchinensis (Lour.) Merr. [Asparagaceae] (Tiandong), Citrus × aurantiifolia (Christm.) Swingle [Rutaceae] (Zhiqiao), and Chrysanthemum arcticum L. [Asteraceae] (Juhua), and is a common formula for the treatment of myopia in Chinese medicine.^[14]^ According to traditional Chinese medicine (TCM), myopia is mostly caused by deficiency of liver and kidney yin and deficiency of Qi and blood, as stated in the TCM classic work Yixue Rumen and approved by modern TCM research^[15]^: “Those who can see near but not far, or see one into two, are deficient in liver and kidney.” Studies on TCM constitutional theory showed that myopia patients were mostly Yin deficiency, and part of them were Qi-stagnation.^[16,17]^ Among the ingredients of DZP, Sheng Dihuang and Tiandong can nourish Yin, Zhiqiao can transform Qi, and Juhua can comfort the liver and brighten the eyes. Therefore, Yin will be replenished, Qi stagnation will be eliminated, and vision will become clear. One study has reported a case of myopia cured by DZP and identified its efficacy in relieving myopia and improving blurred vision.^[14]^ However, the pharmacological mechanisms of DZP in the treatment of myopia are still unclear, which to a certain extent limit the application of DZP in clinic.

The aims of this study are to identify the active constituents, core targets and potential mechanisms of DZP for myopia (including pathways and immune regulation functions), and to assess the stability of the molecular docking of the active constituents of DZP with the intersectional targets and specific targets of myopia. By network analysis and bioinformatics methods, we identified the active constituents and relevant targets of DZP in the treatment of myopia and screened myopia-specific modules and genes. We also explored the potential mechanisms of its action using the Gene Ontology (GO) and Kyoto Encyclopaedia of Genes and Genomes (KEGG) enrichment analyses and differences in immune infiltration levels between groups to verify the immunomodulatory functions of DZP in the treatment of myopia.

## 2. Materials and methods

**Figure 1.**
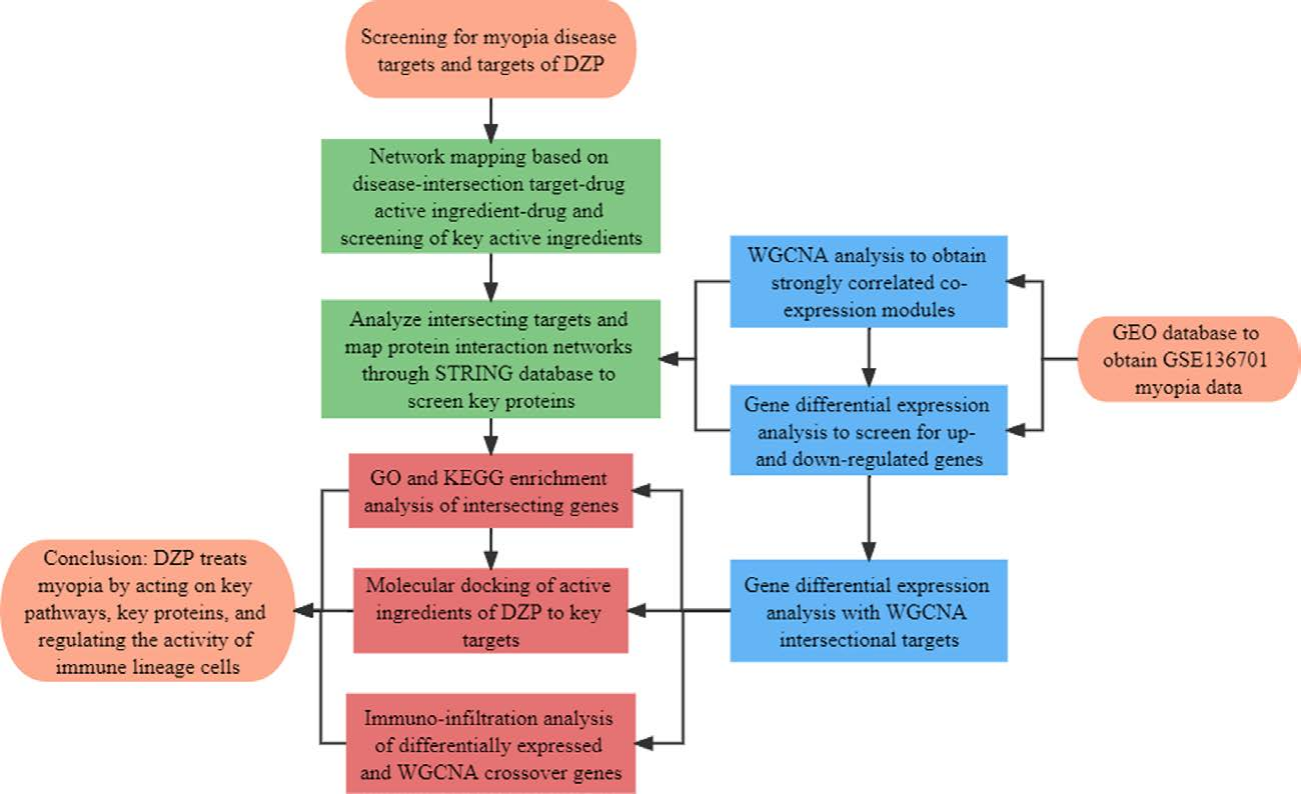
Flow chart of steps used in the exploration of the potential mechanisms of DZP in the treatment of myopia. DZP = Dizhi Pill, GO = gene ontology, KEGG = Kyoto Encyclopaedia of Genes and Genomes, WGCNA = weighted gene co-expression network analysis.

The network analysis materials and methodological steps of this study were performed in accordance with the Guidelines on Network Pharmacology Evaluation Methods.^[18]^ The steps used to identify the mechanisms of DZP for myopia in this study are presented in Figure 1.

### 2.1. Identification of active constituents and related targets of DZP

The active constituents of the ingredients of DZP were searched through the Traditional Chinese Medicine Systems Pharmacology Database and Analysis Platform (TCMSP, https://old.tcmsp-e.com/tcmsp.php) database,^[19]^ and screened according to the standards of oral bioavailability ≥ 30% and drug likeness ≥ 0.18.^[20,21]^ As the ingredient Sheng Dihuang was not included in the TCMSP, the information relevant to its active constituents was searched through the Herb (http://herb.ac.cn/) database, and the studies relevant to its active constituents were searched through China National Knowledge Infrastructure, Wanfang and PubMed databases using the search strategy presented in File S1, Supplemental Digital Content, http://links.lww.com/MD/J488.^[22–24]^ For the active constituents whose oral bioavailability and drug likeness were not available, the SMILES numbers of the active constituents were obtained from the Pubchem (https://pubchem.ncbi.nlm.nih.gov) database, and then the active constituents were screened through Swiss Target ADME (www.swissadme.ch) database according to the Lipinski filter^[25]^ principle and BOILED-Egg^[26]^ principle. After screening of the active constituents, the targets of the active constituents were predicted by the Swiss Target Prediction (www.swisstargetprediction.ch) database, and then the protein names were converted into their standard upstream gene names by the Uniprot (https://www.uniprot.org) database and the duplicated results were removed. These proteins and targets were tagged with their upstream gene names in the following sections of this study.

### 2.2. Identification of myopia-related targets

Myopia-related targets were obtained through the Genecards (https://www.genecards.org), Online Mendelian Inheritance in Man (https://www.omim.org), Drugbank (https://go.drugbank.com),^[27]^ and Therapeutic Targets Database (http://db.idrblab.net) databases using the search term “myopia” and the duplicate results were removed.

### 2.3. Identification of myopia gene expression profiles and analysis of differentially expressed genes

We searched “myopia” in the GEO database to obtain gene expression profile data GSE136701 of lens epithelium in high myopia and normal eyes.^[28]^ Gene ID conversion was conducted using the GPL570 platform to transform the ESEMBLE numbers into the gene symbols. The data was analyzed by the online analysis function GEO2R, and the Differentially Expressed Genes (DEGs) between myopia and normal samples were screened according to the statistical significance of the between-groups difference (*P* < .05), with log_2_FC < -1 as down-regulated genes and log_2_FC > 1 as up-regulated genes.^[29]^ The significant DEGs among groups were selected in order of correlations of their Gene Counts between samples. The DEGs were shown using a heat map and a volcano map created by RStudio and the website “bioinformatics” (www.bioinformatics.com.cn).

### 2.4. Weighted gene co-expression network analysis

Weighted gene co-expression network analysis (WGCNA) was performed on myopia gene expression profile data using the “WGCNA” package of RStudio to screen the characteristic modules and genes of myopia.^[30]^ After clustering analysis to remove outlier gene expression data from each sample, the adjacency between genes was calculated by network topology analysis using the standard of *R*^2^ = 0.7 to screen the best soft threshold.^[31]^ The adjacency was transformed into a topological overlap matrix and the dissimilarity was calculated. The clustering tree diagram was drawn by hierarchical clustering method and the minimum number of module genes was set as 300, division sensitivity as 1, and distance of merging similarity modules as 0.3 to construct the weighted gene co-expression network. In the phenotypic correlation analysis, modules with the strongest positive and negative correlations between modules and the grouping factor were screened and the genes in the modules were selected.

### 2.5. Immune infiltration analysis

The intersectional genes in the results of differentially expressed gene (DEG) analysis and WGCNA were screened and the immune infiltration analysis was performed using the CIBERSORT algorithm.^[32]^ The TPM values of normalized counts values in myopia gene expression profile data were calculated to predict the differences in the proportions of immune cells between myopia and normal samples, and the immunomodulatory functions of the constituent ingredients of DZP on each immune cell in the treatment of myopia were validated by reviewing the correlational studies.

### 2.6. Visualization of intersectional targets

The overlap targets between DZP and myopia and the overlap targets between DEG analysis and WGCNA were screened using Microsoft Excel. The intersecting targets were visualized using the JVenn plot from the Bioinformatics website.^[33]^

### 2.7. Establishment of intersecting target protein-protein interactions (PPI) network

The intersecting targets were imported into the String (https://cn.string-db.org) database and the species was set as “Homo sapiens.” The minimum required confidence score was set as “highest confidence (0.900)” for the analysis of the intersectional targets of DZP and myopia and was set as “medium confidence (0.400)” for the analysis of the intersectional targets of DEG analysis and WGCNA. PPI networks were drawn using Cytoscape_v3.9.1 software based on the analysis results of String database. The degree centrality (DC) of each node protein in the PPI networks was calculated using the function “CytoNCA.”

### 2.8. DZP ingredients-drug-target-disease network construction

The information of active constituents, related targets of DZP and myopia-related targets was collated to establish the interrelationships among DZP ingredients, active constituents, DZP and myopia intersectional targets, and myopia. A DZP ingredients – active constituents – targets – myopia network was plotted using the software Cytoscape_v3.9.1, and DC of each node in the network was calculated using the function “Tools-Network Analyzer.”

### 2.9. Enrichment analysis

To identify which pathways were the most related to the intersectional targets, and to conclude the potential pathways relevant to effect of DZP and myopia development, the enrichment analyses were performed using the “clusterProfiler” package of RStudio. The species was set as “Homo sapiens,” and enrichment analyses were performed based on the GO and KEGG databases according to the selection standard *P* value < .05.^[34]^ Pathways of the KEGG enrichment analysis and pathways in 3 categories of the GO enrichment analysis – molecular function (MF), cellular component (CC), biological process (BP) – were derived and the results of the enrichment analyses were visualized using bubble plots.

### 2.10. Molecular docking

The top 5 DC-ranked active constituents in the results of DZP ingredients – active constituents – targets – myopia network were selected for molecular docking with the top 6 DC-ranked intersectional targets of DZP and myopia, and with the top 2 DC-ranked targets of myopia screened by WGCNA and DEG analysis. Protein structures were obtained by the Uniprot and RCSB PDB (www.pdbus.org) databases, and structures of active constituents were obtained by the Pubchem database. Autodock 1.5.7 was used to perform dehydration, hydrogenation, small molecule ligand energy minimization, acquisition of active pockets and molecular docking of active constituents and targets. According to the official Autodock recommendations, 50 molecular docking operations were performed for each pair of active constituent and target,^[35]^ and the binding activity between each pair of molecule and target was considered good when the molecular docking binding energy was equal or superior to 5 kJ/mol.^[36]^ The results were visualized using PyMOL 2.2.0 software.

### 2.11. Ethical review

In this study, the data analyzed were all sourced from public databases and did not include any personal data, therefore the ethical approval was waived.

## 3. Results

### 3.1. Information of the active constituents of DZP

We identified a total of 43 active constituents of DZP, including 17 from Juhua, 13 from Sheng Dihuang obtained from the Herb database and 3 correlational studies,^[22–24]^ 7 from Tiandong, 3 from Zhiqiao, and 3 duplicate active constituents, among which naringenin (A1) originated from both Juhua and Zhiqiao, quercetin (A2) originated from both Juhua and Tiandong, and beta-sitosterol (B1) originated from all 4 ingredients (File S2, Supplemental Digital Content, http://links.lww.com/MD/J489). There were 412 predicted targets and 1027 additional targets obtained from relevant studies. A total of 758 targets were obtained after de-duplication. The information of the active constituents of DZP sorted according to their action targets is presented in Table 1.

**Table 1:** Ingredients of Dizhi pill, available MOL ID of the active constituents, names of the active constituents, numbers of action targets of active constituents, screening standards according to Swiss Target ADME or oral bioavailability and drug likeness values of the top 4 active constituents of Dizhi pill ingredients.

Ingredients	MOL ID	Active constituents	Targets	Standard
Tiandong	MOL000098	quercetin	266	OB (%) = 46.43, DL = 0.28
	MOL003896	7-Methoxy-2-methyl	92	OB (%) = 42.56, DL = 0.20
	MOL000358	beta-sitosterol	62	OB (%) = 36.91, DL = 0.75
	MOL000449	Stigmasterol	61	OB (%) = 43.83, DL = 0.76
Juhua	MOL000098	quercetin	266	OB (%) = 46.43, DL = 0.28
	MOL000422	kaempferol	124	OB (%) = 41.88, DL = 0.24
	MOL000006	luteolin	101	OB (%) = 36.16, DL = 0.25
	MOL004328	naringenin	86	OB (%) = 59.29, DL = 0.21
Zhiqiao	MOL004328	naringenin	86	OB (%) = 59.29, DL = 0.21
	MOL005828	nobiletin	85	OB (%) = 61.67, DL = 0.52
	MOL000358	beta-sitosterol	62	OB (%) = 36.91, DL = 0.75
	MOL002341	hesperidin	31	OB (%) = 70.31, DL = 0.27
Sheng Dihuang	NA	Diincarvilone A	123	Swiss Target ADME standard
	NA	Ferulic acid methyl ester	105	Swiss Target ADME standard
	NA	Rehmapicrogenin	78	Swiss Target ADME standard
	NA	beta-sitosterol	62	OB ≥ 30%, DL ≥ 0.18

DL = drug likeness, NA = not available, OB = oral bioavailability.

### 3.2. Myopia-related targets

A total of 5393 myopia targets were obtained from the Drugbank, Therapeutic Targets Database Online Mendelian Inheritance in Man and Genecards databases after de-duplication.

### 3.3. DEG analysis of myopia gene expression profiles

**Figure 2.**
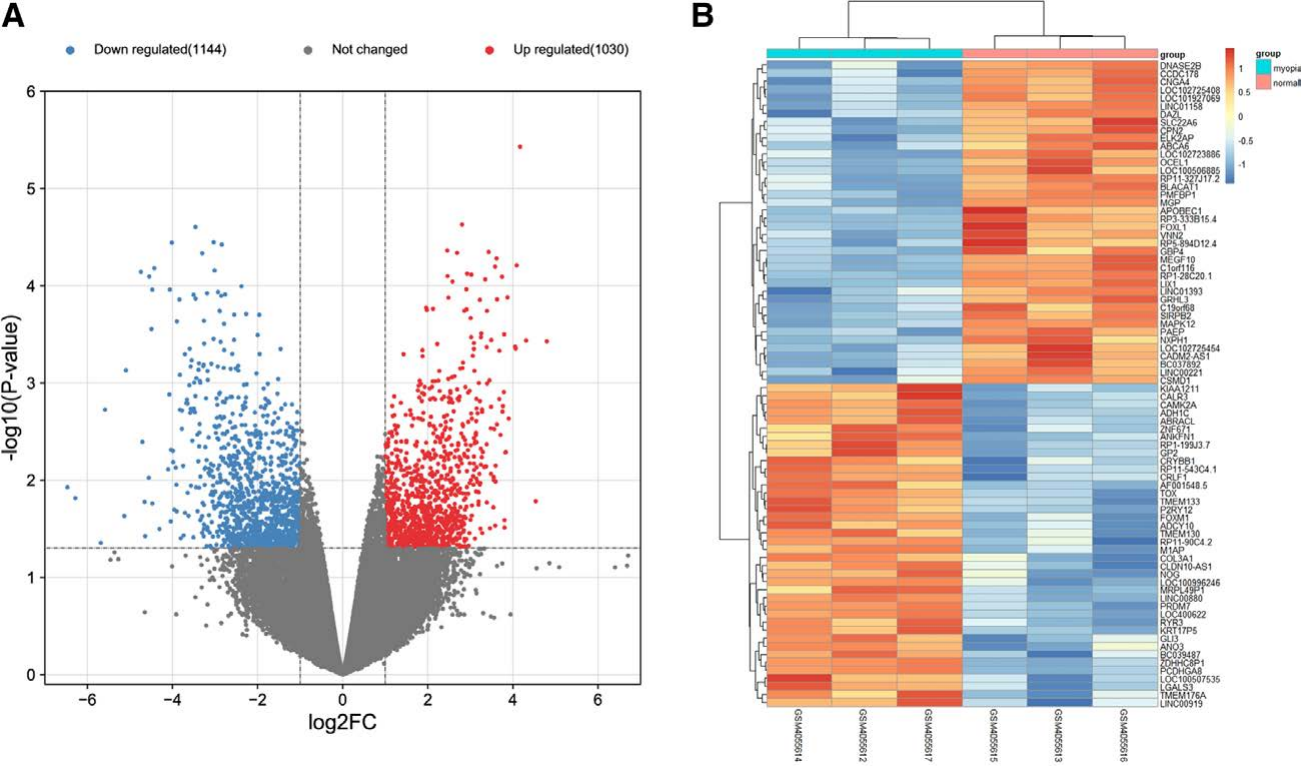
Volcano plot and heat map of the DEGs in the expression profile GSE136701 of myopia. A: Up-regulated genes are displayed in red and down-regulated genes are displayed in blue. B: The first 3 columns represent gene expressions in people with myopia and the last 3 columns represent gene expressions in people who do not have myopia. DEGs = differentially expressed genes.

A total of 23,348 genes were obtained after gene ID conversion using GPL570 platform (File S3, Supplemental Digital Content, http://links.lww.com/MD/J490), including 446 down-regulated genes and 395 up-regulated genes. The volcano plot of DEG analysis is presented in Figure 2A. The 40 most significant genes among groups are displayed in order of correlations of their Gene Counts between samples and are visualized using the heat map in Figure 2B. We found that the significant up-regulated genes in myopia samples were KIAA1211 and CALR3, and the significant down-regulated genes were DNASE2B and CCDC178. These genes may be strongly involved in the development of myopia.

### 3.4. Results of WGCNA

**Figure 3.**
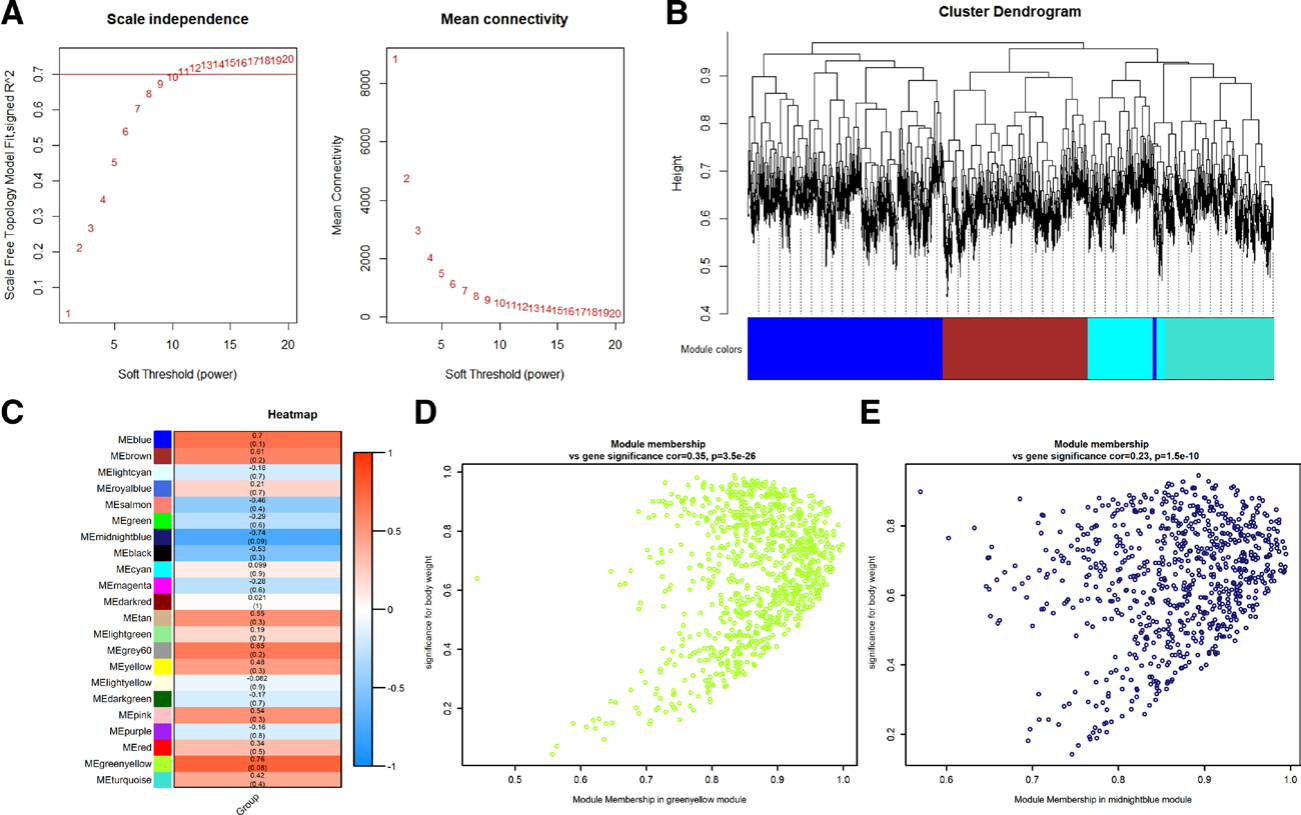
Results of weighted gene co-expression network analysis. A: Selection of the optimal soft threshold using network topology analysis method. B, C: Scatter points plots of gene significance and module membership correlations of genes in the modules. D: Cluster dendrogram constructed by hierarchical clustering method based on the topological overlap matrix of genes, module identification results in 5 parts with the first part of the clustering dendrograms and module colors shown in the figure. E: Heat map of the correlations between 22 co-expression modules and group clinical phenotype.

The weighted gene co-expression network analysis was performed for 23,348 genes of GSE136701 and the best soft threshold 11 was taken to construct the WGCNA network presented in Figure 3A. A total of 22 merged modules with similar expression patterns were screened from the results of the clustering tree (Fig. 3B). The correlations between modules and the grouping factor are displayed in Figure 3C with grouping factor as the clinical phenotype. The 1617 targets in “green yellow” (correlation coefficient = 0.76) and “midnight blue” (correlation coefficient = −0.74) modules with the strongest positive and negative correlations were selected as WGCNA results. The scatter plots of gene significance (GS) and module membership of genes in the 2 modules with grouping factor as the clinical phenotype are presented in Figure 3D and E. The scatter distributions were mainly concentrated in the upper right, which meat that the genes in each module were highly correlated with both the phenotype and modules.

### 3.5. Identification of the intersectional targets

**Figure 4.**
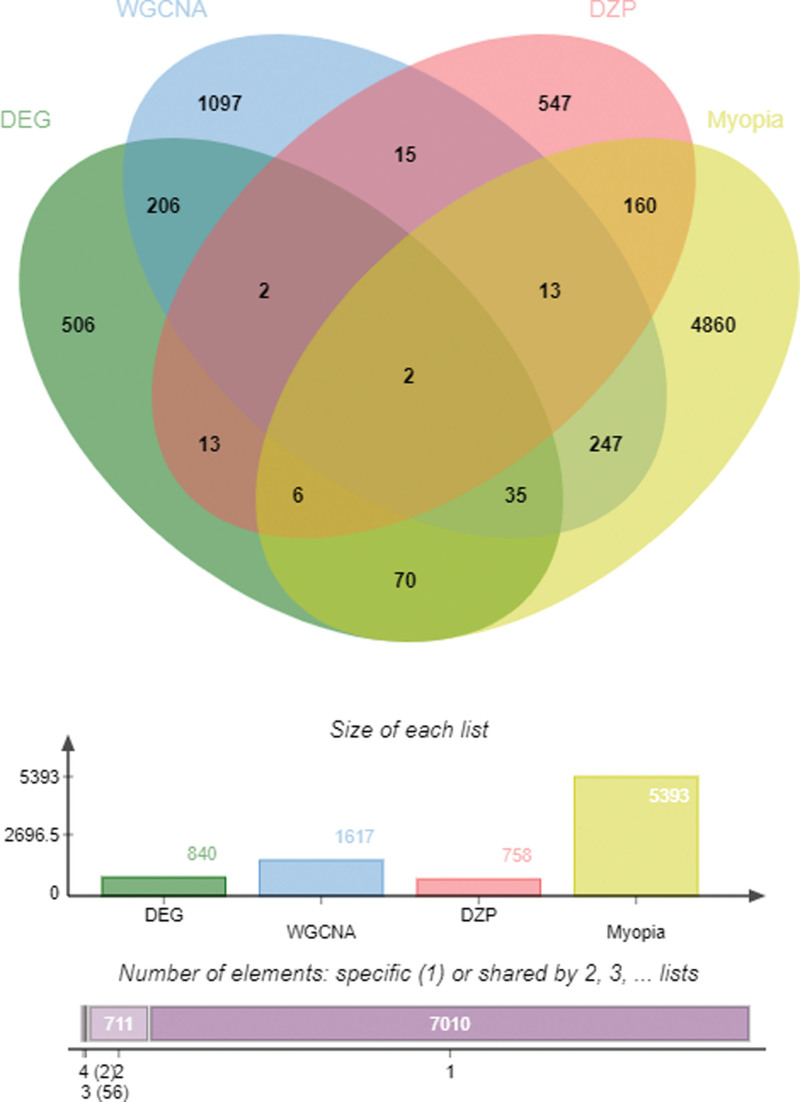
JVeen plot of the intersectional targets of DEG, WGCNA, myopia and DZP. DEG = differentially expressed gene, DZP = Dizhi pill, WGCNA = weighted gene co-expression network analysis.

As mentioned above, we identified 5393 targets related with myopia, 758 potential targets of DZP, 841 targets corresponding to their up-regulated and down-regulated genes in DEG analysis, and 1617 targets of WGCNA results. The interrelationships of these targets are shown in the JVenn plot (Fig. 4). We obtained 181 intersectional targets of DZP and myopia (File S4, Supplemental Digital Content, http://links.lww.com/MD/J491), 245 intersectional targets of DEG analysis and WGCNA, and 2 (AR and TYRO3) intersectional targets of DZP, myopia, DEG analysis and WGCNA. The 181 intersectional targets of DZP and myopia were considered the functional targets of DZP in treating myopia, and 245 intersectional targets of DEG analysis and WGCNA were considered as core targets of myopia which may related to the development and occurrence of myopia.

### 3.6. PPI network construction for intersectional targets

**Figure 5.**
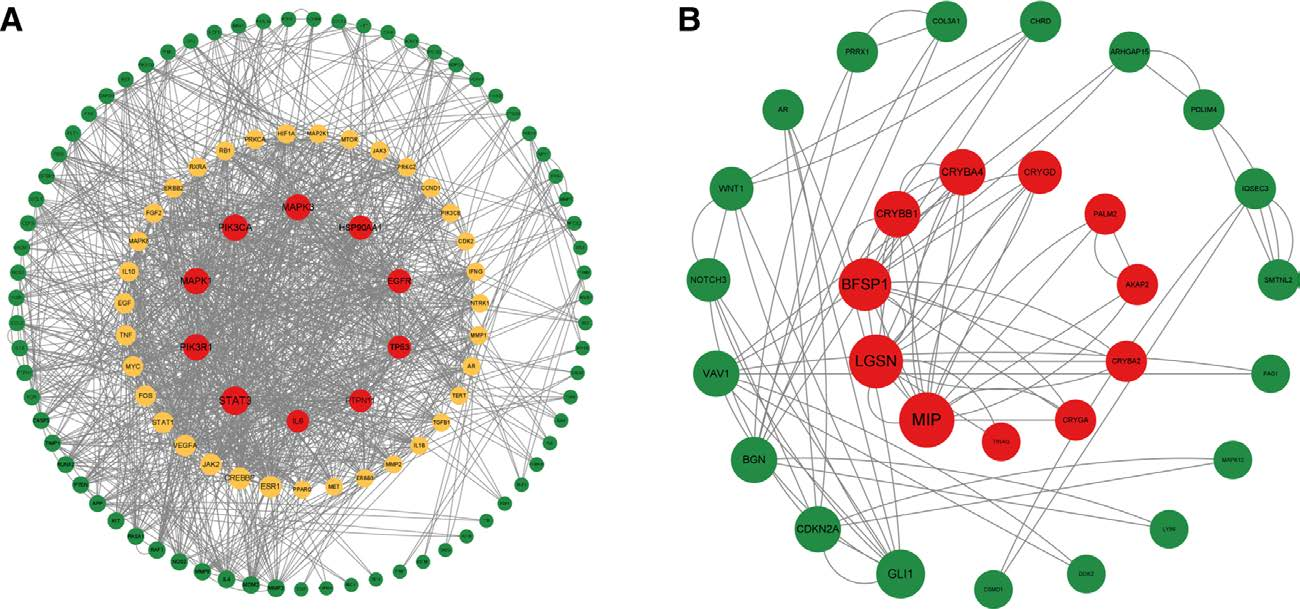
A: PPI network of the intersectional targets between myopia and DZP. B: PPI network of the 245 intersectional targets of DEG analysis and WGCNA. Diameters of the nodes are proportional to the DC values of nodes in the figures and the significant nodes in terms of DC are shown in red. DC = degree centrality, DEG = differentially expressed gene, DZP = Dizhi pill, PPI = protein-protein interactions, WGCNA = weighted gene co-expression network analysis.

The PPI network of the 181 targets that were associated with both DZP and myopia is presented in Figure 5A. After removing the unlinked and locally connected targets, the graph contained 116 nodes and 1216 edges in total. The PPI network of the 245 intersectional targets of DEG analysis and WGCNA is presented in Figure 5B. A total of 30 nodes and 85 edges were obtained after removing targets that could not be retrieved and locally connected targets. The information of the top 5 DC-ranked intersectional targets is presented in Table 2. The detail information of the PPIs is presented in File S5, Supplemental Digital Content, http://links.lww.com/MD/J492, File S6, Supplemental Digital Content, http://links.lww.com/MD/J493, Files S7 and S8, Supplemental Digital Content, http://links.lww.com/MD/J494.

**Table 2:** Network nodes characteristic parameters of the top 5 targets according to degree centrality ranking in the protein-protein interactions networks.

Rank	Targets	DC	Betweenness	Closeness
PPI network for intersectional targets of myopia and DZP				
1	STAT3	84	1658.992395	0.550239234
2	PIK3CA	72	766.9793804	0.504385965
3	PIK3R1	72	719.9914882	0.504385965
4	MAPK1	72	1140.108614	0.534883721
5	MAPK3	70	1076.371843	0.532407407
PPI network for intersectional targets of DEG analysis and WGCNA				
1	MIP	15	38.333332	0.04982818
2	LGSN	14	26.333334	0.04974271
3	BFSP1	13	13.333333	0.04974271
4	GLI1	10	185.000000	0.076719575
5	BGN	8	94.000000	0.07178218

DC = degree centrality, DEG = differentially expressed gene, DZP = Dizhi pill, PPI = protein-protein interactions, WGCNA = weighted gene co-expression network analysis.

### 3.7. DZP ingredients – active constituents – targets – myopia network

**Figure 6.**
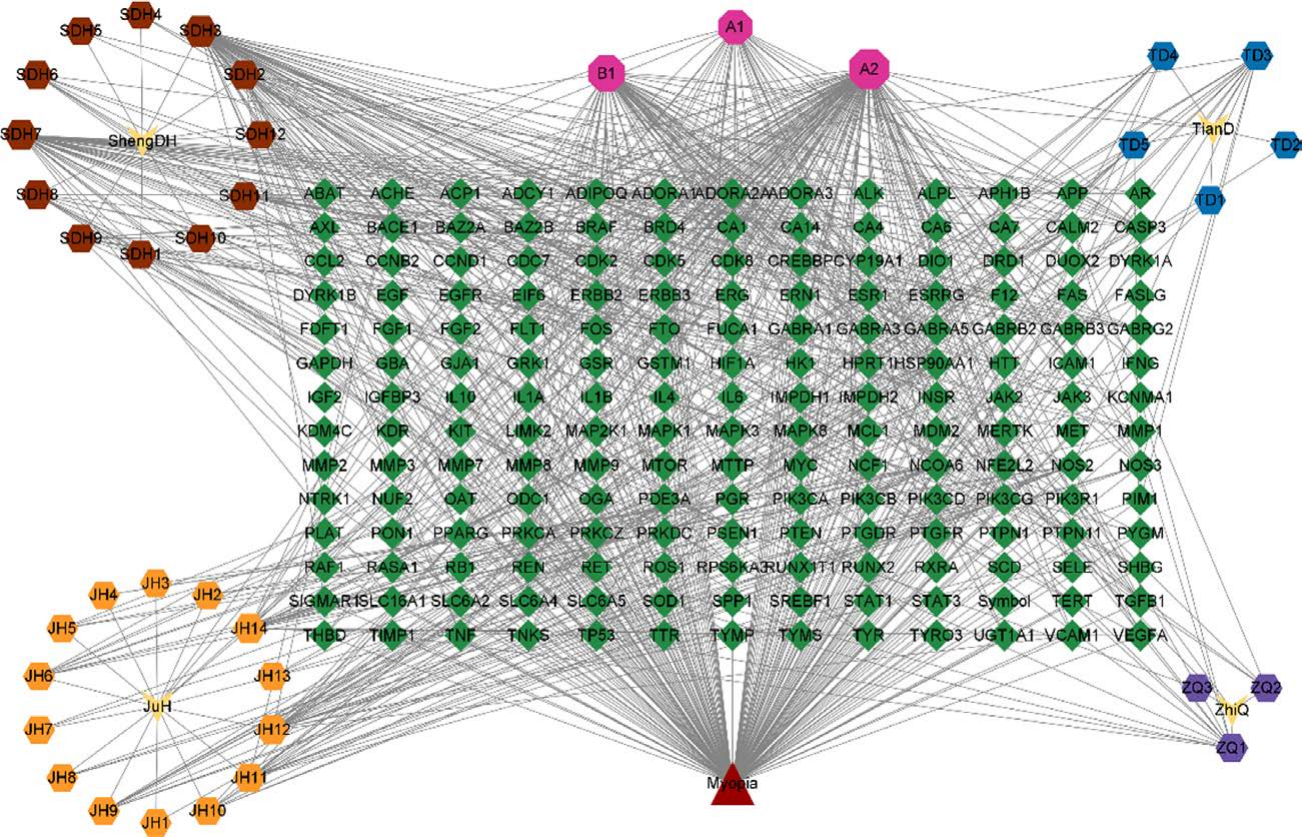
DZP ingredients – active constituents – targets – myopia network. In this network, green modulars represent the targets, yellow modulars represent the DZP ingredients, hexagon modulars represent the active constituents, and triangle modular represents myopia. A1, A2, B1 represent the common active constituents of DZP. DZP = Dizhi Pill.

The network of DZP ingredients – active constituents – targets – disease created with Cytoscape 3.9.1 is presented in Figure 6 and File S9, Supplemental Digital Content, http://links.lww.com/MD/J495. The top 10 active constituents of DZP according to DC ranking are presented in Table 3.

**Table 3:** Network nodes characteristic parameters of the top 10 active constituents according to degree centrality ranking in the Dizhi pill ingredients – active constituents – targets – myopia network.

Rank	Active Constituents	Identifier	DC	Betweenness	Closeness
1	Quercetin	A2	128	0.062291583	0.432170543
2	Beta-sitosterol	B1	68	0.01490572	0.376689189
3	Diincarvilone A	SDH3	59	0.034913112	0.403985507
4	Ferulic acid methyl ester	SDH7	46	0.027125262	0.388501742
5	Naringenin	A1	36	0.008784137	0.363192182
6	Luteolin	JH14	28	0.012467097	0.379251701
7	Kaempferol	JH12	24	0.010006629	0.376689189
8	Rehmapicrogenin	SDH2	23	0.011491382	0.375420875
9	7-Methoxy-2-methyl isoflavone	TD3	16	0.004987216	0.362012987
10	Coniferin	SDH8	15	0.003437673	0.346273292

DC = degree centrality.

### 3.8. Results of the GO and KEGG enrichment analyses

**Figure 7.**
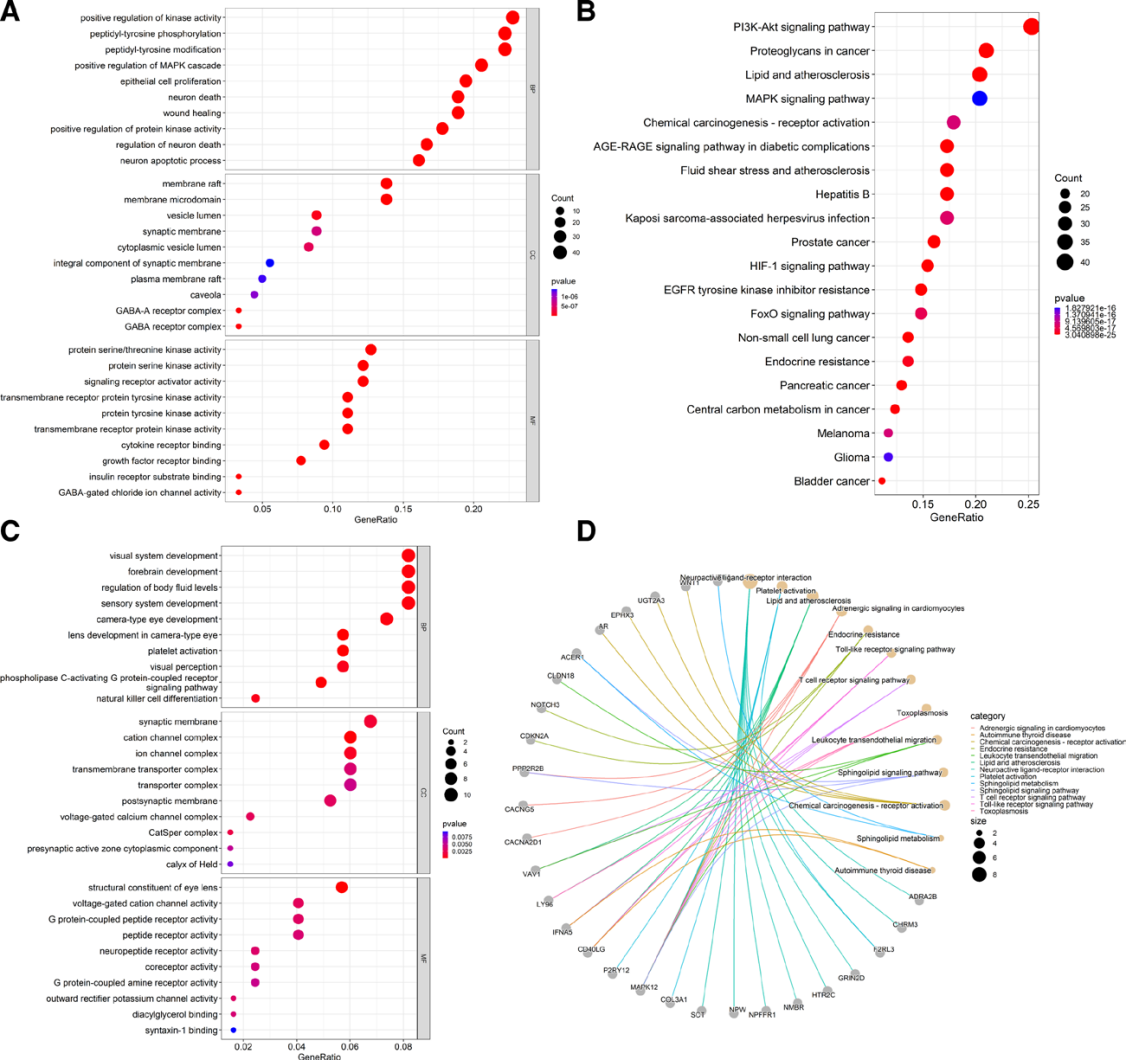
A–D. Results of the GO and KEGG enrichment analyses of DZP in treating myopia. GeneRatio was the ratio of the number of genes on the pathway to the number of genes in the total enriched pathways. Red color represents a smaller *P* value and purple color represents a bigger *P* value. DZP = Dizhi Pill, GO = gene ontology, KEGG = Kyoto Encyclopaedia of Genes and Genomes.

**Figure 8.**
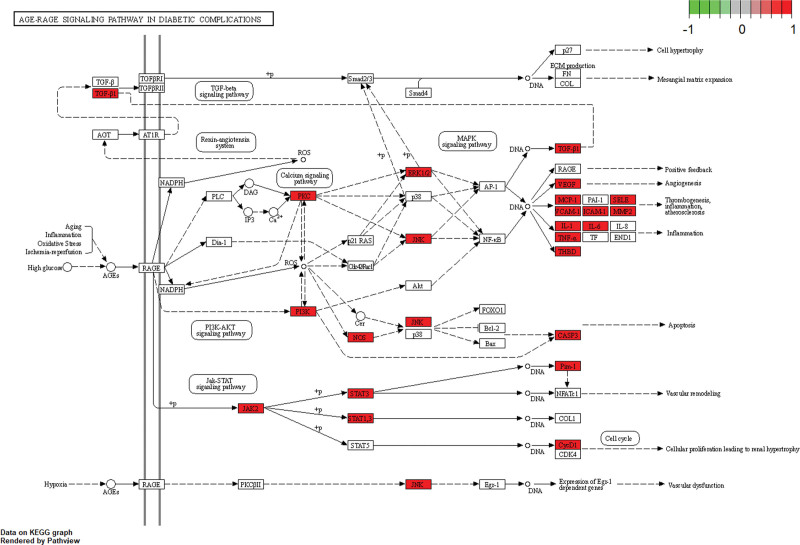
Mechanism of DZP active constituents on myopia through the “AGE-RAGE diabetic complication signaling” pathway. The solid arrows in the diagram represent signaling facilitation, the dashed arrows represent inhibition, the red targets play an up-regulation role on signaling and the green targets play a down-regulation role. AGE-RAGE = advanced glycosylation end-products-Receptor for advanced glycosylation end-products, DZP = Dizhi pill.

A total of 2741 GO-enriched pathways (File S10, Supplemental Digital Content, http://links.lww.com/MD/J496), including 199 pathways in MF, 99 pathways in CC, 2443 pathways in BP, and 171 KEGG pathways (File S11, Supplemental Digital Content, http://links.lww.com/MD/J497), were obtained from the 181 intersectional targets of DZP and myopia. The GO enrichment pathways with the highest number of enriched targets were “positive regulation of kinase activity” and “transmembrane receptor tyrosine kinase activity” pathways (Fig. 7A), while the KEGG enrichment pathways related to myopia were “Advanced Glycosylation End-products to the Receptor for Advanced Glycosylation End-products (AGE-RAGE) signaling pathway in diabetic complications” and “EGFR tyrosine kinase inhibitor resistance” pathways (Fig. 7B). Among these pathways, the “AGE-RAGE signaling pathway in diabetic complications” was the most closely related to the regulation of myopia progression, and the mechanism diagram of “AGE-RAGE diabetes complication signaling” pathway in the regulation of myopia was drawn using the “clusterprofiler” package of RStudio (Fig. 8). The results showed that there were 3 inhibition pathways in the 4 downstream pathways of AGE-RAGE: “TGF-beta signaling,” “renin-angiotensin system” and “PI3K-AKT signaling” pathways, and the targets of DZP were mostly upregulated in these 3 inhibitory pathways. After the enrichment analyses of 245 intersectional targets from WGCNA and DEG analysis, the GO analysis identified 224 pathways in BP, 24 pathways in CC and 34 pathways in MF (File S12, Supplemental Digital Content, http://links.lww.com/MD/J498), and the KEGG analysis identified 13 pathways (File S13, Supplemental Digital Content, http://links.lww.com/MD/J499). Sorted by the numbers of targets on the pathways, the GO results were mainly related to “lens structural composition,” “cation channel complex” and “platelet activation” pathways (Fig. 7C) while KEGG results were mainly related to “neuroactive ligand receptor interactions” and “lipid and atherosclerotic” pathways (Fig. 7D). The detail information of the enrichment pathways is presented in the supplementary files “GO” and “KEGG.”

### 3.9. Results of immune infiltration analysis

**Figure 9.**
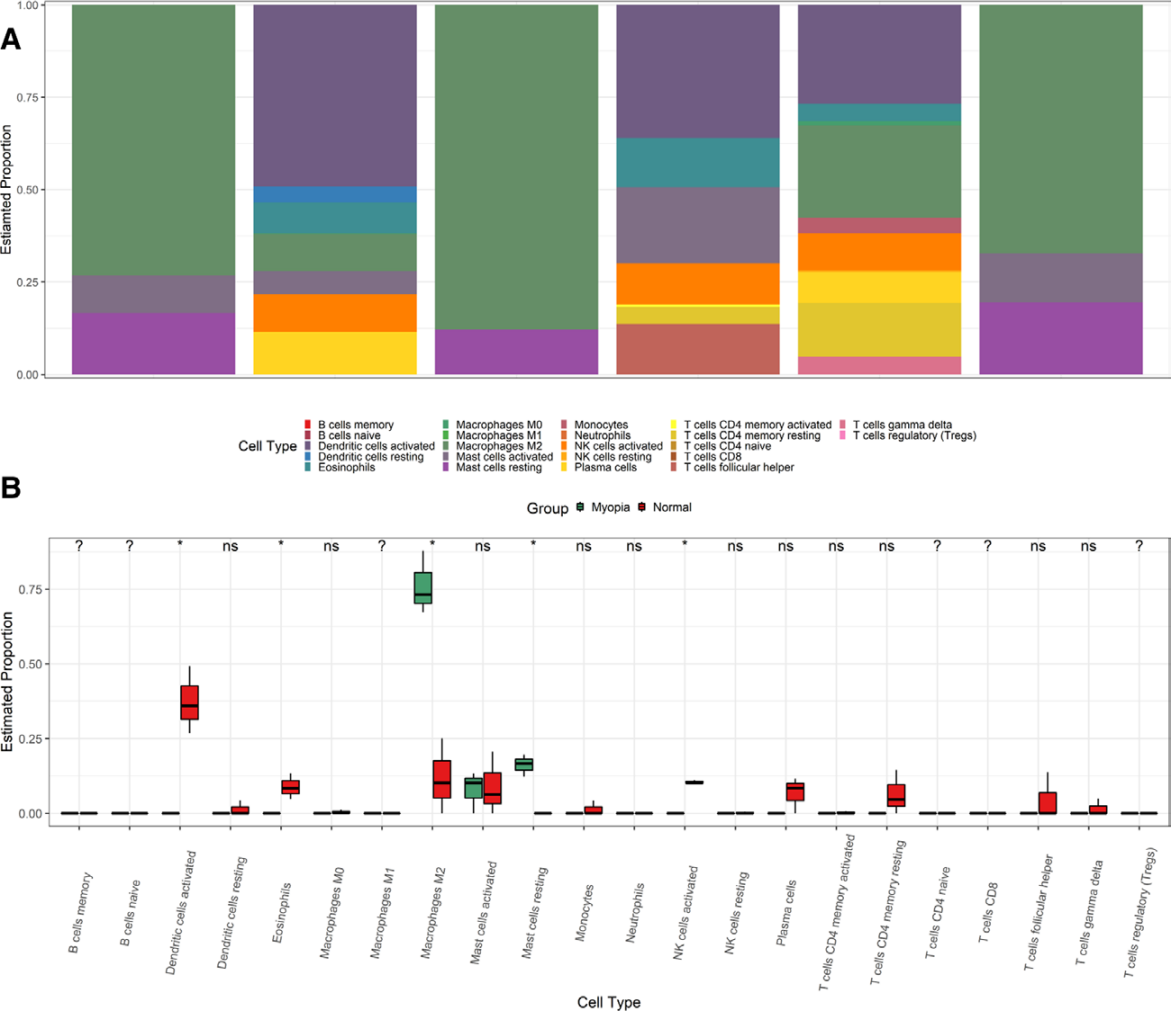
Immune infiltration analysis of the intersectional genes of WGCNA and DEG analysis. A: Histogram of the percentages of immune cells in each sample. B: Box line plot of the percentages of various immune cells between myopia (in green) and non-myopia individuals (in red). DEG = differentially expressed gene, WGCNA = weighted gene co-expression network analysis.

The results of the immune infiltration analysis are presented in Figure 9A and B. The immune infiltration results showed the increased proportions of inactivated mast cells, M2 macrophages in high myopia lens samples, and decreased proportions of eosinophils, activated dendritic cells, NK cells, plasma cells, activated mast cells and CD4 T cells in high myopia lens samples.

### 3.10. Molecular docking

**Figure 10.**
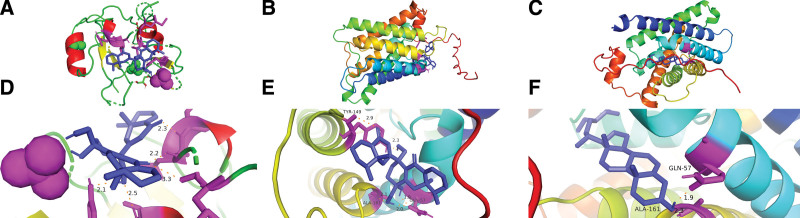
A–F. Molecular docking results of the active constituents Diincarvilone A and Beta-sitosterol with targets HSP90AA1 and MIP. The purple and pink modules in D, E and F represent the active constituents and protein binding sites, respectively.

The important intersectional targets: STAT3, PIK3CA, PIK3R1, MAPK1, MAPK3, HSP90AA1, MIP and LGSN in Table 2 were molecularly docked with the top 5 active constituents (quercetin, Beta-sitosterol, Diincarvilone A, Ferulic acid methyl ester and naringenin). The binding energies are presented in Table 4, where the drug small molecules could spontaneously and firmly bind to the target proteins. The firmest 3 dockings are displayed, including Diincarvilone A binding to amino acids GLN-329, LEU-328, MWT-326, VAL-401 and TYR390 of target PIK3R1 through hydrogen bonds (Fig. 10A and D), with a binding energy of -38.158 kJ/mol, Diincarvilone A binding to amino acids TYR-149, GLN-57 and ALA-161 of target MIP through hydrogen bonds (Fig. 10B and E), with a binding energy of −36.233 kJ/mol, and Beta-sitosterol binding to amino acids GLN-57 and ALA-161 of target MIP through hydrogen bonds (Fig. 10C and F), with a binding energy of −34.518 kJ/mol.

**Table 4:** Binding energies between core targets and important active constituents (kJ/mol).

Active constituents	Targets							
	STAT3	PIK3CA	PIK3R1	MAPK1	MAPK3	HSP90AA1	MIP	LGSN
Quercetin	−22.133	−31.464	−26.568	−26.234	−26.819	−28.409	−32.552	−19.288
Beta-sitosterol	−29.372	−33.974	−32.635	−31.422	−31.506	−32.384	−34.518	−27.196
Diincarvilone A	−27.112	−29.999	−38.158	−24.895	−28.911	−30.920	−36.233	−25.522
Ferulic acid methyl ester	−18.075	−23.681	−22.301	−21.673	−20.878	−23.179	−22.175	−18.326
Naringenin	−24.560	−25.648	−26.234	−29.079	−28.660	−30.376	−24.686	−23.138

## 4. Discussion

In this study we identified 5 active constituents of DZP which had the highest number of functional targets, 6 significant intersectional targets and 2 core targets of myopia which had the highest DC in PPI. A total of 597 pathways were identified which were related to the mechanisms of DZP in treating myopia. Additionally, we identified 4 significant DEGs of myopia: KIAA1211, CALR3, DNASE2B, and CCDC178, and 2 common targets: AR and TYRO3 among DZP, myopia, DEG analysis, and WGCNA. The combinations of molecules and targets in molecular docking were stable, and our results showed that the immune regulatory functions of DZP ingredients could regulate the abnormal proportions of immune cells in people with myopia.

Active constituents involved in the therapeutic effects of DZP on myopia mainly included quercetin, β-sitosterol, Diincarvilone A, methyl ferulate, and naringenin. Quercetin is a flavonol that can be processed into ophthalmic ointment preparations and has good efficacy in the treatment of metabolic cataract.^[37]^ Animal experimental studies have demonstrated that quercetin could effectively inhibit the expression of transforming growth factor (TGF-β1) in corneal tissue thereby slowing down corneal scar formation and promoting corneal tissue recovery in alkali-burned rats,^[38]^ which had a protective effect on vision. It has been demonstrated that anti-vascular endothelial growth factor (VEGF) drugs could inhibit VEGF receptor activity and thus reduced the activity of the dopamine (DA) system and disrupted the balance between D1 and D2 receptors,^[39]^ and then the imbalanced DA system could cause prolongation of the eye axis and increased myopia.^[40]^ β-sitosterol can activate the KDR gene, which is highly associated with its function, and can activate the VEGF receptor encoded by KDR gene which can increase and adjust DA levels in the retina so as to achieve the purpose of treating myopia.^[41]^ In addition, studies have pointed out that the lack of ocular blood perfusion could exacerbate myopia, and retinal vessel diameter and blood perfusion were greater in patients with milder myopia.^[42,43]^ At the same time, intraocular inflammatory factors could promote the exacerbation of myopia and lead to retinal lesions in patients.^[44]^ Existing pharmacological studies in Chinese medicine have demonstrated that the active constituents of DZP, such as β-sitosterol, umbelliferyl and isohesperidin, had anti-inflammatory and immunomodulatory effects as well as functions of vasodilating and improving blood circulation,^[45–48]^ which may help to restore eye blood perfusion, alleviate eye verification and thus treat myopia, but the specific mechanisms remain to be clarified.

The 6 important intersectional targets of DZP for myopia treatment were STAT3, PIK3CA, PIK3R1, MAPK1, MAPK3, and HSP90AA1. In recent years, STAT3 has gradually gained attention in the field of ophthalmology. One study found the increased expression of activated STAT3 in the retina of FDM guinea pigs, suggesting that STAT3 may be involved in the formation and development of myopia.^[49]^ As a signaling protein, it could act on the photoreceptor outer segments of the retina,^[50]^ and continuous chronic stimulation from STAT3 could cause retinal lesions and aggravate myopia.^[51]^ HSP90AA1 is a heat shock protein (HSP) and its functions are related to immunity, structural maintenance and repair. The expression of α-Crystallin, a major component of the lens which also belongs to HSP, can interact with degeneration proteins to inhibit degeneration protein aggregation and therefore maintain the size and transparency of the lens^[52]^ and can reduce the number of degeneration proteins to avoid the formation of severe myopia.^[28]^

The enrichment analyses of GO and KEGG found that the pathways closely related to DZP and myopia targets were “AGE-RAGE signaling pathway in diabetic complexes” and “positive regulation of ATP metabolic process” pathways. The AGE-RAGE signaling pathway can regulate the binding of advanced glycosylation end-products (AGE) to the receptor of advanced glycosylation end-products (RAGE), which can further activate Muller glial cells so as to promote VEGF secretion.^[53]^ The overexpressed VEGF can promote the excessive proliferation of retinal blood vessels, which will lead to the development of low myopia to high myopia, and even lead to retinal structural damage, macular degeneration, cataract, etc.^[54]^ According to the result of Figure 8, it showed that the targets of DZP played an upregulation role in 3 inhibitory signals downstream of the AGE-RAGE pathway, leading to the speculation that DZP may inhibit the deterioration and progression of myopia through the inhibition of AGE-RAGE signal pathway. ATP is a cell activator, which can act on tired ciliary muscle cells to relieve their spasm, and also has an atropine-like effect to dilate the pupil, which can be dropped in the eye to treat pseudo myopia.^[55]^ Therefore, DZP may increase ATP level by the “positive regulation of ATP metabolic process” pathway so as to treat myopia.

The results of cross-target enrichment analyses by WGCNA and DEG analysis showed that the pathways closely related to myopia mainly involved “platelet activation” and “neuroactive ligand receptor interactions.” Studies have shown that asparagus, a constituent of DZP, had the effect of reducing platelet aggregation.^[56]^ In addition, Juhua can reduce platelet activation derivative TXA2 and platelet adhesion rate, which can repair abnormal platelet activation function.^[57]^ In one study, after the administration of asparagus decoction to SD rats, neuroactive enzyme levels in the brain tissue of SD rats increased and acetylcholine transferase levels decreased, showing a neuroactive role.^[58]^ The studies mentioned above may help to support our hypothesis that the effect of DZP in treating myopia is achieved through the “platelet activation” and “neuroactive ligand receptor interactions” pathways.

Our study showed that the proportions of inactivated mast cells and M2 macrophages increased and the proportions of activated dendritic cells, activated mast cells, plasma cells, and CD4 T cells were lower in people with myopia. One study showed that after the administration of DZP to mice, the number of Langerhans dendritic cells in the skin as well as their number of dendrites increased.^[59]^ Juhua can inhibit the activation and phagocytosis of macrophages,^[60]^ and asparagus can increase the proportions of CD4T cells and plasma cells.^[61]^ Based on the immune regulatory effects of the constituent ingredients of DZP and the immune infiltration results of myopia samples, it is predicted that DZP may play an active role in regulating myopia.

Although the downstream targets of key myopia genes predicted by WGCNA and DEG analysis were not among the predicted targets of DZP, the molecular docking results showed that MIP and LGSN could firmly bind to the active constituents of DZP. MIP could bind to Dincarvillone A with a binding energy of −36.233 kJ/mol, which was second only to the stability of PIK3R1 and Dincarvillone A in 40 docking groups. The results of molecular docking suggested that the active constituents of DZP could effectively and stably combine with the myopia core targets, which may provide supports for further research to confirm the therapeutic effect of DZP for treating myopia.

This study revealed the potential mechanisms of DZP for myopia through diverse analyses and fastidious methodology. However, the results of immune infiltration and bioinformatic analyses cannot provide direct evidence for the efficacy of DZP on myopia. Randomized-controlled trials are required to evaluate its efficacy directly. Presently, there are no randomized controlled trials on DZP for myopia, so it is not available to provide systematic evaluation evidence. The active constituents identified by this study, quercetin and beta-sitosterol, are widespread polyphenols, known for nonspecific in silico effects and act as pan-assay interfering substances. Therefore, the conclusions of these 2 substances should be treated with caution, and whether they can play a role in the treatment of myopia also needs to be further verified by subsequent research.

Based on the network analysis and bioinformatic analyses, this study provided a noninvasive and moderate treatment for myopia, filled the gap in the research of the mechanisms traditional Chinese medicine in treating myopia, and provided guidance for the future research direction on the pharmacological effects of DZP in treating myopia. DZP may exert its efficacy by regulating the DA system, balance between D1 and D2 receptors and the levels of VEGF and ATP in the eyes. Clinically, low or middle myopia generally does not cause great pathological damages to patients, but high myopia can bring a variety of pathological damages, such as macular degeneration, cataract, and retinal detachment, etc.^[62]^ DZP can be used as an auxiliary treatment for the pathological damages of myopia, especially in the treatment of high myopia, because it can inhibit the progression of macular degeneration and cataract through the function of anti-VEGF, and its function of improving ATP level can alleviate the lengthening of ocular axis and the degeneration of eyeball caused by ciliary muscle spasm, thus preventing the occurrence of retinal detachment in high myopia. In addition, clinicians are suggested to properly adopt Dincarvilone A to treat myopia, because its strong interactions with other intersectional targets and high binding energies with the core targets of myopia. When treating myopia patients with diabetes, more attention can be paid to the regulation of AGE-RAGE pathway. Subsequent studies can further study the pharmacological effects of DZP on myopia and develop new drugs according to the targets and pathways revealed in this study. This study has proved the effects of DZP on the basis of bioinformatics and network analyses, which can provide reference for the follow-up studies to conduct randomized-controlled trials of DZP in the treatment of myopia.

In summary, based on the network analysis and bioinformatics analyses, this study suggested that the active constituents of DZP could act on intersectional targets: STAT3, PIK3CA, PIK3R1, MAPK1, MAPK3, and HSP90AA1, core targets of myopia: MIP and LGSN, “AGE-RAGE signaling” and “positive regulation of ATP metabolic process” pathways to treat myopia. DZP active constituents and myopia targets could bind stably and immune infiltration analysis showed the potential mechanisms for the efficacy of DZP.

## Author contributions

**Conceptualization:** Longkun Liu, Yan Zhao, Li Li, Zhaolan Liu.

**Data curation:** Longkun Liu.

**Formal analysis:** Longkun Liu, Li Li, Zhaolan Liu.

**Funding acquisition:** Li Li, Zhaolan Liu.

**Investigation:** Zhaolan Liu.

**Methodology:** Longkun Liu, Yoann Birling, Yan Zhao, Wenxin Ma, Yang Tang, Yuxin Sun, Xuehui Wang, Mingkun Yu, Li Li, Zhaolan Liu.

**Software:** Longkun Liu, Zhaolan Liu.

**Writing – original draft:** Longkun Liu, Yoann Birling, Yan Zhao, Wenxin Ma, Yang Tang, Yuxin Sun, Xuehui Wang, Mingkun Yu, Hongsheng Bi, Jianping Liu, Li Li, Zhaolan Liu.

## Correction

This article was published with errors in the contact information for the corresponding author. The correct contact information is “*Correspondence: Li Li, Beijing Institute for Drug Control, NMPA Key Laboratory for Safety Research and Evaluation of Innovative Drugs, Beijing Key Laboratory of Analysis and Evaluation on Chinese Medicine, Beijing, China (lili_bidc@163.com).”

## Supplementary Material
























