# Supplementary material for: Mechanism of Chinese botanical drug Dizhi pill for myopia: An integrated study based on bioinformatics and network analysis
Source: Medicine (Baltimore). 2023 Sep 22;102(38):e34753. doi: 10.1097/MD.0000000000034753 (PMC10519534; doi:10.1097/MD.0000000000034753)
Supplement: Supplementary file 1 [file medi-102-e34753-s001.pdf]

Search strategy in PubMed, CNKI and Wanfang

Items	Search Terms
Search strategy in PubMed	
1	Rehmannia glutinosa [Title/Abstract] OR Chinese Foxglove [Title/Abstract] OR Shengdihuang [Title/Abstract] OR Sheng Dihuang [Title/Abstract]
2	chemical composition [Title/Abstract] OR Active ingredient [Title/Abstract] OR Active constituents [Title/Abstract]
4	1 AND 2
Search strategy in CNKI	
1	TI=('生地黄'+'生地'+'地黄')
2	TI=('研究进展'+'研究'+'进展'+'作用机理'+'实验研究')
3	TI=('化学成分'+'结构鉴定'+'化学成分研究'+'研究进展'+'药理活性'+'药理作用')
4	1 AND 2 AND 3
5	KY=('生地黄'+'生地'+'地黄')
6	KY=('研究进展'+'研究'+'进展'+'作用机理'+'实验研究')
7	KY=('化学成分'+'结构鉴定'+'化学成分研究'+'研究进展'+'药理活性'+'药理作用')
8	5 AND 6 AND 7
9	4 OR 8
Search strategy in Wanfang	
1	题名或关键词=("生地黄" or "生地" or "地黄")
2	题名或关键词=("研究进展" or "研究" or "进展" or "作用机理" or "试验研究")
3	题名或关键词=("化学成分" or "结构鉴定" or "化学成分研究" or "研究进展" or "药理活性" or "药理作用")
4	1 AND 2 AND 3

CNKI: China National Knowledge Infrastructure.
