# Supplementary material for: Mechanism of Chinese botanical drug Dizhi pill for myopia: An integrated study based on bioinformatics and network analysis
Source: Medicine (Baltimore). 2023 Sep 22;102(38):e34753. doi: 10.1097/MD.0000000000034753 (PMC10519534; doi:10.1097/MD.0000000000034753)
Supplement: Supplementary file 7 [file medi-102-e34753-s007.pdf]

Infromation of the PPI diagram of intersectional targets of Dizhi pill and myopia

Targets	Degree	Betweenness	Closeness
ACHE	2	0	0.266821346
APP	18	1123.815046	0.362776025
ADCY1	2	0	0.270588235
IL1A	16	230.2094069	0.36977492
ADIPOQ	8	45.16116035	0.356037152
CREBBP	46	699.836506	0.456349206
PPARG	22	173.1812266	0.419708029
CDK8	8	19.95945589	0.308310992
RXRA	30	496.5473738	0.447470817
ALK	4	0	0.345345345
PIK3CA	72	766.9793804	0.504385965
JAK3	28	109.6189334	0.444015444
APH1B	4	0	0.26744186
PSEN1	6	1	0.268065268
GAPDH	12	153.5270351	0.391156463
TTR	2	0	0.266821346
CASP3	18	260.0204161	0.403508772
MAPK8	32	558.0595888	0.454545455
NTRK1	26	583.5833315	0.447470817
BACE1	4	0	0.26744186
AR	26	324.9506021	0.450980392
CCND1	28	166.0196301	0.444015444
MDM2	22	90.22157188	0.415162455
BRD4	6	1.015873016	0.357142857
STAT3	84	1658.992395	0.550239234
RB1	30	159.3067509	0.438931298
EGFR	58	478.1352245	0.497835498
HSP90AA1	60	535.4301628	0.493562232
RUNX2	18	13.70898502	0.406360424
SHBG	2	0	0.311653117
PIK3R1	72	719.9914882	0.504385965
BRAF	10	0.865079365	0.36977492
MAPK1	72	1140.108614	0.534883721
RAF1	20	29.48240048	0.416666667
MAPK3	70	1076.371843	0.532407407
PRKCA	30	277.006968	0.440613027
MAP2K1	28	220.5559271	0.442307692
MYC	38	366.0559117	0.487288136
TP53	54	767.5962401	0.487288136
CALM2	4	1.427215297	0.310810811
NOS2	20	183.7017855	0.435606061

NOS3	14	71.67960016	0.37704918
MCL1	14	23.52401275	0.429104478
SREBF1	14	81.12491038	0.378289474
PRKDC	8	1.647499559	0.36977492
CCL2	14	2.209406887	0.368589744
FOS	40	414.1981705	0.467479675
IL4	22	59.72970467	0.402097902
IL10	34	160.2563047	0.407801418
TNF	36	596.3425316	0.442307692
IL1B	24	95.96749994	0.421245421
IL6	50	401.2623068	0.463709677
CCNB2	8	672	0.303430079
CDK2	26	1003.315761	0.410714286
NUF2	4	452	0.235173824
CDK5	14	310.1381339	0.387205387
PIM1	10	0	0.389830508
JAK2	44	178.9510383	0.456349206
HIF1A	30	77.71609192	0.44921875
ESR1	46	518.9650228	0.489361702
CDC7	2	0	0.291878173
PTGDR	6	4.643430254	0.347432024
TERT	24	215.8948348	0.435606061
FDFT1	8	0.976190476	0.342261905
NFE2L2	6	3.171527432	0.359375
VEGFA	44	358.1530942	0.463709677
NCOA6	8	7.541587641	0.358255452
HTT	8	4.63392913	0.384615385
STAT1	42	155.0870082	0.473251029
CYP19A1	2	0	0.329512894
EGF	36	97.16992912	0.444015444
FGF2	32	181.299904	0.442307692
KIT	18	7.624342945	0.406360424
RASA1	18	35.13219775	0.392491468
PIK3CB	28	42.30277963	0.392491468
PTPN1	16	6.337432161	0.375816993
MTOR	28	194.969405	0.440613027
PTPN11	52	254.4437488	0.479166667
ERBB2	32	46.31820848	0.445736434
ERBB3	24	15.04903199	0.404929577
PTEN	18	114.9765791	0.395189003
MET	22	30.30775156	0.435606061
FAS	12	9.851616138	0.387205387
IGFBP3	14	104.1941886	0.413669065
PGR	14	2.463916639	0.421245421

ERN1	2	0	0.307486631
FASLG	10	6.805881832	0.382059801
FGF1	4	0	0.350609756
TIMP1	18	82.51259937	0.380794702
MMP2	24	65.1680647	0.402097902
TGFB1	24	97.32516942	0.425925926
KDR	16	13.06511251	0.387205387
FLT1	14	10.48966507	0.378289474
MMP1	26	193.0433313	0.395189003
MMP3	22	463.7193478	0.400696864
MMP9	20	1.682525253	0.384615385
GJA1	4	0	0.350609756
GSTM1	2	0	0.225048924
SPP1	6	230.0857019	0.289672544
IFNG	26	76.20806086	0.425925926
ICAM1	8	190.0897278	0.368589744
SELE	4	0	0.275779376
VCAM1	8	68.58854551	0.32122905
IGF2	10	24.69605708	0.346385542
INSR	14	29.73806818	0.368589744
PRKCZ	28	170.6120384	0.437262357
PIK3CG	12	2.077708733	0.370967742
NCF1	10	82.73722782	0.375816993
RPS6KA3	4	0	0.350609756
RET	12	9.27921094	0.383333333
MMP7	6	9.39512987	0.2875
PIK3CD	6	0	0.351681957
TYMS	4	228	0.191347754
TNKS	4	0.666666667	0.315934066
SCD	4	0	0.315934066
TYMP	2	0	0.160839161

PPI: Protein-protein interactions.

Infromation of the PPI diagram of intersectional targets of DEG analysis and WGCNA

Targets	Degree	Betweenness	Closeness
PAG1	2	0	0.072864322
TINAG	2	0	0.048986486
SMTNL2	4	64	0.070048309
DOK2	2	0	0.072864322
IQSEC3	4	34	0.067599068
CSMD1	2	0	0.065022422
WNT1	6	130	0.075520833
NOTCH3	6	12	0.074935401
MAPK12	2	0	0.071078431
COL3A1	4	0	0.069047619
LY96	2	0	0.06888361
PRRX1	4	0	0.069047619
CHRD	4	112	0.073791349
BGN	8	94	0.071782178
LGSN	14	26.33333333	0.04974271
CRYBA4	8	0	0.049488055
CRYGA	4	0	0.049235993
CRYBA2	4	0	0.049152542
CRYGD	6	0	0.049403748
CRYBB1	8	0	0.049488055
BFSP1	13	13.33333333	0.04974271
VAV1	8	176	0.076115486
PDLIM4	4	90	0.072319202
ARHGAP15	4	112	0.074358974
CDKN2A	8	35	0.074168798
GLI1	10	185	0.076719577
AR	4	0	0.073791349
PALM2	4	0	0.049152542
MIP	15	38.33333333	0.049828179
AKAP2	4	0	0.049152542

PPI: Protein-protein interactions. DEG: Differentially Expressed Gene. WGCNA: Weighted Gene Co-expression Network Analysis.
